# The Prevalence of Disordered Eating Behaviours (DEBs) among Adolescent Female School Students in Riyadh, Saudi Arabia: A Cross-Sectional Study

**DOI:** 10.3390/nu16020281

**Published:** 2024-01-17

**Authors:** Azzah Alsheweir, Elizabeth Goyder, Samantha J. Caton

**Affiliations:** 1 Sheffield Centre for Health & Related Research (SCHARR), School of Medicine & Population Health, University of Sheffield, Sheffield S1 4DA, UK; e.goyder@sheffield.ac.uk (E.G.); s.caton@sheffield.ac.uk (S.J.C.); 2 Department of Community Health Sciences, College of Applied Medical Sciences, King Saud University, Riyadh 145111, Saudi Arabia

**Keywords:** adolescents, disordered eating behaviours, eating disorders, age, weight, BMI, region, EAT-26, Saudi Arabia

## Abstract

Adolescence is a sensitive stage of life that is particularly vulnerable to nutritional problems, including DEBs. This cross-sectional study aims to explore the prevalence of DEBs among adolescent female school students in four intermediate and secondary schools in the city of Riyadh and to examine predictors associated with DEBs, including age, BMI and school regions. A total of 416 female students aged 12–19 years took part in this study. Weight and height were measured for students before the EAT-26 test was completed. Overweight and obesity were observed among 37.7% (*n* = 157) of students, 50.7% (*n* = 211) had a normal BMI and 11.5% (*n* = 48) were underweight. Results indicated that 123 (29.6%) students reported an EAT-26 score of 20 or more, indicating a high risk of DEB. Age was a significant predictor of DEB risk (OR = 3.087, 95% CI = 1.228–7.760), with the older age group (16–19 years) reporting a higher risk than the younger age group (12–15 years) (*p* = 0.017). DEB risk partially differed by school region, but BMI was not a statistically significant predictor. The high-risk group reported more binging (*p* = 0.008), induced vomiting (*p* < 0.001), laxative consumption (*p* < 0.001) and exercising (*p* < 0.001) compared with the low-risk group. Further research is warranted to understand DEB current patterns and predictors.

## 1. Introduction

Disordered eating behaviours (DEBs) are behaviours and attitudes acknowledged as a part of the eating disorder (ED) spectrum [1]. Such behaviours (binge eating, dietary restriction, self-induced vomiting, diet and hunger-repressive pills, diuretics and laxatives) are practiced by approximately 10–20% of adolescent females in Western countries, with estimates reaching 40% for some behaviours [2,3]. Micali and colleagues identified that the peak age of onset for such behaviours is between 15 and 19 years of age among females [4]. These behaviours may result in the development of a fully diagnosable eating disorder, such as binge-eating disorder (BED), anorexia nervosa (AN) or bulimia nervosa (BN), which can have major psychological and physiological complications [5,6].

Adolescence is seen as a particularly nutritionally susceptible stage of life [7,8]. The substantial increase in physical growth and development, as well as the shift in lifestyle and eating habits of adolescents, all contribute to an increased demand for nutrients that affects both nutrient intake and requirements [7]. Restricting energy consumption can be concerning for adolescents, as it may lead to binge eating, irritability, impaired growth and sexual maturation and an elevated risk of DE [8].

Adolescent females are identified to be more prone to DEBs compared with males or other age groups [9]. Particularly, these behaviours are commonly considered the most prevalent health problem associated with obesity in female adolescents [9,10]. A multi-dimensional assessment of disordered eating and body dissatisfaction found that Asian females exhibited more body dissatisfaction and DEB than American females [11]. In other studies concerned with measuring the risk of DEB among adolescents in Arab countries, the risk of DEB in Arab female adolescents was found to be similar to that reported in Western countries and twice as high as reported in South-East Asian countries [12,13,14,15,16].

BMI and shape concerns can have a direct or indirect association with DEB [17]. Evidence indicates that weight gain contributes to elevated body concerns and dieting behaviours in adolescents [18,19,20]. Weight control behaviours and shape concerns increase the possibility of the onset and persistence of ED [21,22,23]. It is imperative to understand that shape concerns and weight control behaviours may act as mediators between BMI and ED symptoms [17].

Studies that focused on DEB prevalence rates and age have varied greatly depending on the methodology used and the sample analysed [24]. Early research indicates that female adolescents engage in more DEBs as they age since they tend to make more social comparisons related to shape and appearance [25,26,27]. A meta-analysis assessed the level of DEB in children and adolescents aged 7 to 18 years from 16 different countries, including Saudi Arabia, and found that more practices of disordered eating were reported with increasing age [28]. The findings from our systematic review, focusing specifically on Saudi Arabia, were also consistent with the age pattern and DEB, as it was noted that older university students had more DEBs (29.4–65.5%) compared with school students (10.2–42.5%) [29].

Regional variances and DEB were distinctive in the literature by Western and non-Western classifications [30]. Only a few research studies have explored the relationship between urban region residence and DEB, acknowledging that the connection between urbanisation and ED is complex rather than causal and likely to vary across contexts/populations [31]. It should be noted that Saudi Arabia has undergone fast economic expansion over the last four decades, accompanied by a dramatic improvement in living standards and the adoption of a “Westernised” lifestyle characterised by unhealthy eating habits and less physical activity [32]. Our systematic review indicated that the prevalence ranged from 29.4% to 65.5% in the eastern region compared with 48.1% in the northwestern region [29]. The northern regions had the lowest prevalence (25.4%) [29]. This is an increasingly important public health concern globally, and surveys in Arab countries, including Saudi Arabia, confirm this concern in the context of increasing overweight and obesity in both adult and school-age populations and high levels of DEB in school-age populations [28,29].

Despite the significant prevalence of DEB, few studies have been carried out in Arab nations [33]. The city of Riyadh, the capital of Saudi Arabia, was selected for this study to explore the prevalence of DEB among adolescent females in intermediate and secondary schools, as we identified a lack of research (with only one other study conducted there more than 20 years previously). That study identified that already 15.9% of female students from intermediate and secondary schools in Riyadh reported DEB [34]. We know from studies in other parts of Saudi Arabia and other Arab countries that prevalence was likely to have increased since that study and therefore a further study was warranted [29]. This piece of research presents a novelty in the world of scientific evidence, as it provides an in-depth exploratory analysis of EAT-26 scores, its subscales and the behavioural questions embedded among female adolescents in a city that has never been thoroughly explored regarding DEB. Therefore, the present cross-sectional study is an exploratory study aiming to assess the prevalence and identify predictors of DEB among adolescent female school students in intermediate and secondary schools in Riyadh. The objectives addressed in this study include: (1) identifying the prevalence of DEB in Saudi female adolescents; (2) measuring the prevalence of unhealthy weight (under- and overweight); (3) examining the differences in EAT-26 scores and variations in age groups, BMI categories and school regions; (4) identifying the differences in behavioural questions according to variations in age groups, BMI categories, EAT-26 categories (low risk/high risk) and school regions; (5) examining the correlation between EAT-26 subscales (dieting scale, oral control scale and bulimia and food preoccupation scale) with age and BMI.

## 2. Materials and Methods

### 2.1. Study Design and Setting

This cross-sectional study included self-administered surveys and anthropometric measurements. It was implemented in intermediate and secondary governmental schools in the city of Riyadh (the capital and largest city in Saudi Arabia) in March/April 2023. Ethical approval was provided by the Research Ethics Committee at the University of Sheffield (Reference Number 050493) and the Research Ethics Committee at King Saud University in Riyadh (No. E-23-7551), with adherence to the principles outlined in the Declaration of Helsinki 1975.

### 2.2. Participants and Recruitment

In Saudi Arabia, the education system is regulated by the Ministry of Education (MoE) and the Education Evaluation Commission (EEC). In the education system in Saudi Arabia, the formulated school years start with elementary (6–11 years), then intermediate (12–14 years), followed by secondary school (15–17 years). Intermediate education comprises three years (grades seven, eight and nine), and secondary education consists of three years (grades ten, eleven and twelve). Schools were selected from different regions in the city of Riyadh to detect differences in DEB and region. All selected schools were governmental schools, so the students were representative of the local population. Four schools were selected from the northern, western and eastern regions to ensure a more representative sample by including all regions of Riyadh. However, no schools were selected from the southern regions, since the majority of students in this area are non-Saudis. Population projections from mid-2021, as reported by the General Authority of Statistics, showed that non-Saudis made up 36.4% of the overall population [35]. A total of 416 female students, aged 12–19 years, were recruited from the following schools: the 128 Secondary School (North) and the 213 Intermediate School (North) in AsSahafa district, the 59 Secondary School (East) in ArRawda district and the 40 Intermediate School (West) in AsSuwaidi. Girls with chronic diseases, such as hypertension, diabetes and hyper/hypothyroidism, or diagnosed with an eating disorder were excluded.

In order to estimate the minimum number of participants to estimate the prevalence of DEB and associated behaviours, the sample size was calculated using Open Epi software, Version 3 for epidemiologic statistics (Supplementary Material File S1). It was accessed through the CDC website as a statistical calculator for cohort and cross-sectional designs [36,37]. The smallest number of participants required to estimate the prevalence of DEB calculated at a 95% confidence level was 207 students, assuming a prevalence of 16% [34]. However, 416 students were recruited to explore differences in DEB prevalence between age groups and BMI categories.

### 2.3. Procedure

After receiving a formal letter from the MoE with the schools’ names enclosed, the schools were visited and parental consent forms were delivered. Collaborating with school heads and teachers, students gave their parents an information sheet that explained the nature of this study, data collected, tools/methods used and an informed consent form that asked parents’ permission for their daughters’ participation in this study by signing the form and returning it to school. It was highlighted that all data collected would be anonymised and only used for this research study and no individual data would be shared by the researcher. Recruited students were invited to join an introductory session guided by the main researcher. During this session, this study was explained in full and student participants were given a participant information sheet and consent form that they were required to complete before partaking in the research. The data collection process in this study included anthropometric measurements and self-reported questionnaires. After collecting the signed consent forms from students, they were called individually for anthropometric measurements, including measuring the students’ weight and height. The Eating Attitudes Test (EAT-26) was then distributed to students in an available classroom. They were reminded about the objectives of this study and the confidentiality of the acquired data.

### 2.4. Anthropometric Data

Weight was measured in kilograms (kg) to the nearest 0.1 kg using an electronic weighing scale (Salter Ultimate Accuracy, Bilston, UK). The scale was calibrated and checked prior to the data collection session. A portable stadiometer (Seca 217 Mobile Stable Stadiometer-Portable Height Measurement Rod) measured height in centimetres (cm) to the nearest 0.1 cm. Students were asked to take off their shoes and empty their pockets of any heavy items. To maintain confidentiality, students were asked to stand backward on the weighing scale, and a piece of paper was attached below the reading (as a shield) to limit students’ ability to view the reading.

BMI was calculated using the reported reading by following the Quetelet equation: BMI = weight (kg)/height (m^2^). The WHO guidelines were used, as these are commonly used in similar populations, and BMI was classified as underweight (BMI < 18.5 kg/m^2^), normal weight (18.5–24.9 kg/m^2^), overweight (25.9–29.9 kg/m^2^) and obese (≥30 kg/m^2^) [34,38,39,40,41,42,43,44].

### 2.5. Questionnaire: Eating Attitudes Test (EAT-26)

The EAT-26 is a validated tool that has been used for evaluating DEB [45,46]. It consists of 26 questions subdivided into 3 subscales: dieting (13 items), bulimia/food preoccupation (5 items) and oral control (7 items). Questions have 6-point Likert-scale responses, with scores ranging from 0 to 3 (“Never”, “Rarely” and “Sometimes”: 0; “Often”: 1; “Very often”: 2; “Always”: 3), with question 26 scored conversely (“Often”, “Very often” and “Always”: 0; “Sometimes”: 1; “Rarely”: 2; “Never”: 3). Scores range from 0 to 78, and a score ≥ 20 indicates DEB [45]. Five additional behavioural questions related to food habits in the last six months are found at the end of the questionnaire. Four questions are related to the frequency of binge eating, self-induced vomiting, using laxatives and exercising, with answers: never, once a month or less, 2–3 times a month, once a week, 2–3 times a week and once a day or more. The fifth question is a yes/no question related to losing 9 kg or more in the last six months.

The Arabic version of EAT-26 was used as it has been previously validated as a highly sensitive tool for ED screening [38,39,41,43,47]. This tool has high criterion-related validity and internal consistency (α = 0.90) validated for the Saudi population [48]. A pilot study was implemented on ten students prior to data collection to ensure validity. Reliability analysis was implemented to determine the validity of the Arabic EAT-26 test used in this study and the test showed good internal consistency and reliability (α = 0.774).

### 2.6. Data Analysis

Data were entered and analysed using the Statistical Package for the Social Sciences software SPSS (version 29). Descriptive statistics were presented as means, standard deviations, frequencies and percentages. For research purposes and to facilitate comparisons between study categories, students were grouped into younger (12–15 years) and older (16–19 years) groups to reflect the general demographics in intermediate versus secondary school. Participants were also grouped according to their EAT-26 scores into a high-risk (EAT-26 ≥ 20) and a low-risk group (EAT-26 < 20) [45,46]. A regression analysis was used to investigate the differences in scores among age, BMI and schools (regions). Responses for EAT-26 subscales and behavioural questions were reported as means and standard deviations for the total sample of students. A Chi-square test (χ^2^) was used to determine the differences in responses across age groups, BMI groups and EAT-26 categories. A Pearson correlation was used to evaluate the link between the continuous scores of EAT-26 subscales with age and BMI. A *p*-value of <0.05 was considered statistically significant.

## 3. Results

### 3.1. Demographic Characteristics

A total of 416 students took part in this research, selected from four governmental intermediate and secondary schools in the city of Riyadh. The mean age of all students was 14.56 years (SD = 1.89), with a minimum age of 12 and a maximum of 19 years. The mean BMI was 22.56 kg/m^2^ (SD = 4.92). Half of the students had a normal BMI (*n* = 211, 50.7%), followed by overweight students (*n* = 102, 24.5%). Fifty-five students were obese (13.2%) and forty-eight were classified as underweight (11.5%). Other demographic data are presented in Table 1.

### 3.2. EAT-26 Scores

Across the study population, 123 students (29.6%) reported a score of 20 or more, indicating a high risk of DEB. Three-quarters (*n* = 293, 70.4%) of the study population scored lower than 20, reporting a low risk of DEB.

To determine the prevalence of DEB risk across age groups, BMI categories and school regions, a binary logistic regression was used. Table 2 demonstrates the distribution of age, BMI and school region across EAT-26 categories. Age significantly predicted DEB risk (OR = 3.087, 95% CI = 1.228–7.760), with a higher risk reported among the older age group compared with the younger age group (*p* = 0.017). BMI was not a statistically significant predictor of DEB. However, students in the obese category (38.2%) reported the highest risk, followed by students in the normal weight category (29.4%). For variations in EAT-26 scores among schools, only the 213 Intermediate School in the north (OR = 3.109, 95% CI = 1.016–9.509, *p* = 0.047) and the 40 Intermediate School in the west (OR = 3.071, 95% CI = 1.016–9.282, *p* = 0.047) showed significant difference compared with the reference group.

### 3.3. EAT-26 Subscales (Dieting, Oral Control and Bulimia and Food Preoccupation)

According to students’ responses on the dieting subscale from the EAT-26 test, the higher means were reported for statements that related to burning calories while exercising (1.33 ± 1.31), followed by the fear of being overweight (1.07 ± 1.25) and preoccupation with the desire to be thinner (1.06 ± 1.26). Responses to oral control subscale statements revealed that displaying self-control around food had the highest mean (1.37 ± 1.23), followed by thoughts from other people of being too thin (0.92 ± 1.24) and taking longer than others to eat meals (0.72 ± 1.11). Based on the bulimia and food preoccupation subscale, feelings that food controls life (0.50 ± 0.93) had the highest mean, followed by being preoccupied with food (0.38 ± 0.79) (Table 3).

### 3.4. Behavioural Questions

For behavioural question responses, Table 4 shows the frequency of binge eating, induced vomiting, using laxatives, exercising and losing weight among students. Approximately 4% of students (*n* = 16) reported having binge episodes once or more per day and 3.6% (*n* = 15) reported doing so two to six times per week. In the previous six months, about 9% of students claimed to have experienced two or more induced-vomiting episodes. Thirteen students (3.1%) reported consuming laxatives to control weight two times or more and 173 students (41.5%) claimed to work out for 60 min two times or more in the last six months in an effort to reduce or maintain their weight. In terms of weight loss in the previous six months, approximately 15% of students (*n* = 61) reported dropping 9 kg.

### 3.5. Behavioural Questions (QA Binge Eating, QB Vomiting, QC Laxatives, QD Exercising) and Age

**Figure 1:**
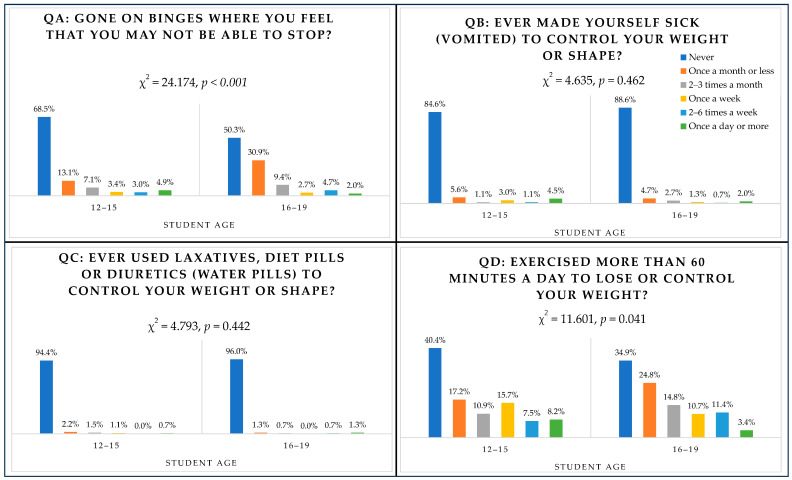
Responses to behavioural questions according to age group.

Figure 1 shows students’ responses by age group. Among behaviours, binge eating and exercising differed significantly across age groups. In relation to binge eating, 8 younger students reported binge eating two to six times a week (3%) and 13 reported binge eating once a day or more (4.9%) versus 7 (4.7%) students and 3 (2%) students from the older group, respectively (χ^2^(5) = 24.174, *p* < 0.001). Exercising more than 60 min per day differed significantly, with more students in the younger group exercising two to six times a day (*n* = 20, 7.5%) compared with students in the older group (*n* = 17, 11.4%), and more students in the younger group (*n* = 22, 8.2%) exercising once a day or more compared with students in the older group (*n* = 5, 3.4%) (χ^2^(5) = 11.601, *p* = 0.041).

### 3.6. Behavioural Questions (QA Binge Eating, QB Vomiting, QC Laxatives, QD Exercising) and BMI

**Figure 2:**
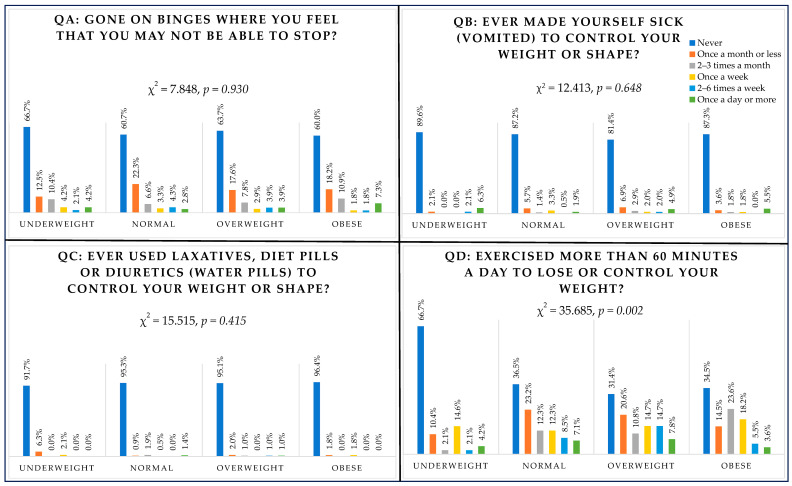
Responses to behavioural questions according to BMI group.

Only exercising was associated with the BMI category (χ^2^(15) = 35.685, *p* = 0.002) (Figure 2). Students in the overweight category reported the highest percentage for those exercising two to six times a week (14.7%) and once a day or more (7.8%) in the previous six months, followed by students in the normal weight category, with 8.5% exercising two to six times a week and 7.1% exercising once a day or more. Among those classified as obese, 5.5% reported exercising two to six times a week and 3.6% reported exercising once a day or more compared with 2.1% and 4.2% of students in the underweight category.

### 3.7. Behavioural Questions (QA Binge Eating, QB Vomiting, QC Laxatives, QD Exercising) and EAT-26 Scores (High Risk/Low Risk)

**Figure 3:**
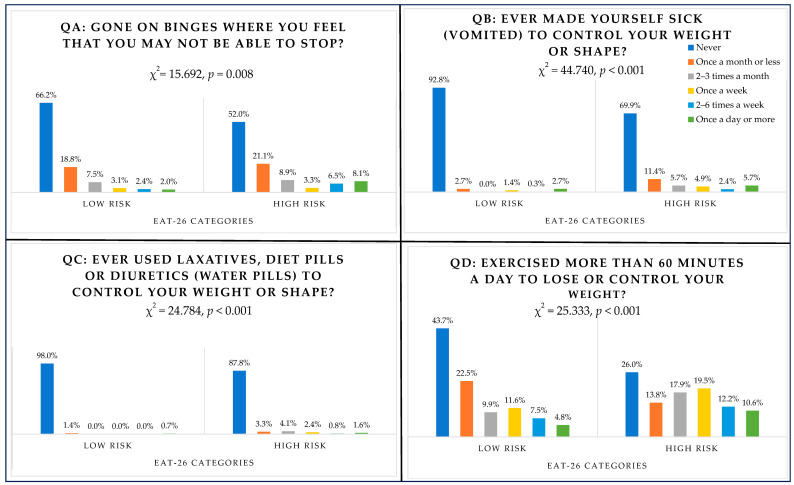
Responses to behavioural questions according to EAT-26 categories.

As shown in Figure 3, responses to behavioural questions were categorised according to EAT-26 scores. All behavioural questions revealed significant differences between high-risk and low-risk groups. Binging was greater among the high-risk group, which scored 20 or more on the EAT-26 test, with 3.3% (*n* = 4) binging once a week, 6.5% (*n* = 8) of students binging two to six times a week and 8.1% (*n* = 10) binging once a day or more (χ^2^(5) = 15.692, *p* = 0.008). Induced vomiting was reported once a week by six high-risk students (4.9%) (χ^2^(5) = 44.740, *p* < 0.001). Three high-risk students reported inducing vomiting two to six times a week (2.4%) and seven students reported it once a day or more (5.7%). Consuming laxatives appeared to be the least common behaviour experienced by students, with only three high-risk students (2.4%) using laxatives once a week, one student (0.8%) using them two to six times a week and two students (1.6%) consuming them once a day or more (χ^2^(5) = 24.784, *p* < 0.001). Exercising for 60 min or more for the sake of losing weight was reported once a week by 19.5% of high-risk students (*n* = 24), 12.2% (*n* = 15) exercised two to six times a week and 10.6% (*n* = 13) exercised once a day or more (χ^2^(5) = 25.333, *p* < 0.001).

Figure 1 shows students’ responses by age group. Among behaviours, binge eating and exercising differed significantly across age groups. In relation to binge eating, 8 younger students reported binge eating two to six times a week (3%) and 13 reported binge eating once a day or more (4.9%) versus 7 (4.7%) students and 3 (2%) students from the older group, respectively (χ^2^(5) = 24.174, *p* < 0.001). Exercising more than 60 min per day differed significantly, with more students in the younger group exercising two to six times a day (*n* = 20, 7.5%) compared with students in the older group (*n* = 17,11.4%) and more students in the younger group (*n* = 22, 8.2%) exercising once a day or more compared with students in the older group (*n* = 5, 3.4%) (χ^2^(5) = 11.601, *p* = 0.041).

### 3.8. Behavioural Question E (Weight Loss) and Age, BMI, EAT-26 Scores (High/Low Risk)

**Figure 4:**
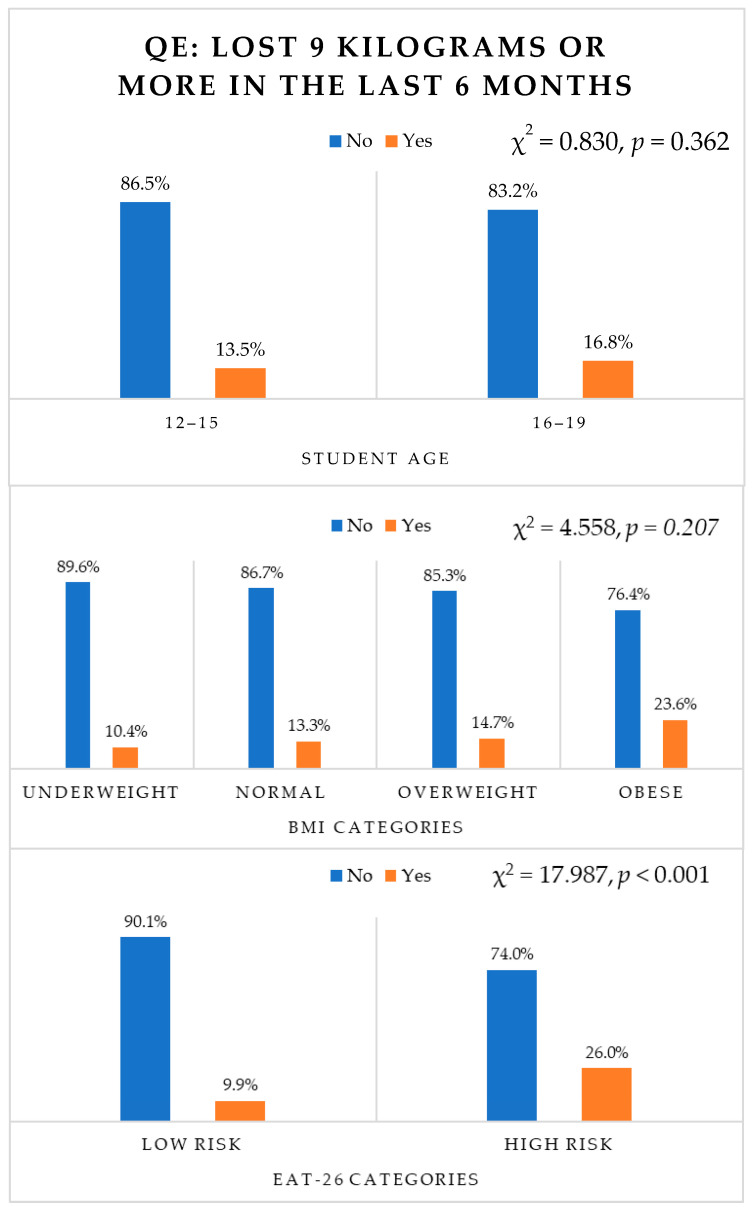
Responses to behavioural question E according to age, BMI and EAT-26 categories.

As shown in Figure 4, weight loss did not differ significantly across age groups (*p* = 0.362) and BMI categories (*p* = 0.207). However, weight loss varied significantly with EAT-26 categories (χ^2^(1) = 17.987, *p* < 0.001). Out of the high-risk group, 32 students (26%) reported losing 9 kg or more in the last six months.

### 3.9. Behavioural Questions and Schools (Regions)

In relation to students’ responses to behavioural questions across schools, none of the behaviours showed significant differences, except for binge eating (χ^2^(15) = 35.556, *p* = 0.002). The proportion of students who reported binge eating once a day or more ranged from 3% to 7% within schools, with the highest percentage reported by students from the 40 Intermediate School in the west (*n* = 7, 7%), followed by the 128 Secondary School in the north (*n* = 2, 3.2%). The proportion of students who had binge-eating episodes 2–6 times a week ranged from 2% to 4.4% across schools, with the highest percentage reported from the 213 Intermediate School in the north (*n* = 6, 4.4%) followed by the 59 Secondary School in the east (*n* = 5, 4.3%).

### 3.10. Correlation between EAT-26 Subscales with Age and BMI

Pearson correlation was used to evaluate the associations between the EAT-26 dieting scale, oral control scale and bulimia and food preoccupation scale with age and BMI [33]. As shown in Table 5, age was not associated with any of the EAT-26 subscales. BMI had a positive association with the dieting scale (*p* < 0.001) and a negative association with oral control (*p* < 0.001).

## 4. Discussion

This is the first study that explores Saudi female adolescents in the city of Riyadh and their DEBs using the EAT-26 test by assessing each individual’s behaviour (binging, induced vomiting, consuming laxatives, intensive exercising and losing weight) and exploring variations with age, BMI, EAT-26 scores (high/low risk) and regions employing a comprehensive analysis of results. The main objectives highlighted in this research include identifying the prevalence of DEB; measuring the prevalence of unhealthy weight (under- and overweight); examining the variations of EAT-26 scores in relation to age, BMI and school regions; identifying the differences in behavioural question responses according to age groups, BMI categories and EAT-26 categories (high/low risk); and determining the correlation between EAT-26 subscales (dieting scale, oral control scale and bulimia and food preoccupation scale) with age and BMI.

Overall, the results of the current study showed that the prevalence of DEB in Saudi female adolescents is 29.6%. Unhealthy weight (under- and overweight) prevalence was 24.7%, with 55 students categorised as obese (13.2%) and 48 students categorised as underweight (11.5%). According to the EAT-26 scores, only age was identified as a significant predictor of DEB, with a higher risk reported among the older age group compared with the younger age group. For behavioural questions, binging and exercising showed significant variations among the older and younger groups, with older girls demonstrating more binging and exercising compared with younger girls. Exercising was the only behaviour that varied significantly across BMI categories, with girls categorized as overweight demonstrating the highest levels of exercising, followed by the obese category. Significant variations were noted among high- and low-risk groups concerning all behaviours—binging, induced vomiting, consuming laxatives, exercising and losing weight—with the high-risk group demonstrating more engagement. The tested correlation between EAT-26 subscales with age and BMI found that only BMI was positively associated with the dieting scale and negatively associated with oral control.

One significant finding was that 29.6% of female school students in Riyadh were categorised as a high risk of DEB, attaining a score of 20 or more on the EAT-26. This rate was significantly higher than the 15.9% reported by Al-Subaie, who recruited female students from intermediate and secondary schools in Riyadh [34]. However, Allihaibi concluded that the prevalence was 26.1% among female secondary students in Makkah (western region of Saudi Arabia), which was only slightly lower than our findings [40]. On the other hand, Al-Qahtani and Al-Harbi identified a higher prevalence of such behaviours in female secondary students in Madina (western region of Saudi Arabia), at 42.5% [41]. These variations in prevalence rates could be due to methodological differences between studies, populations and settings. Specifically, the tool that was employed may have contributed to the variations in the research findings in Riyadh; for example, while we used the EAT-26, Al-Subaie employed the EDI-DT [34].

Age appeared to be a significant predictor of DEB, with older adolescents at a higher risk compared with younger adolescents. This was consistent with earlier and recent findings that confirmed that the prevalence and intensity of DEB increased with age [2,25,26,27,28,49]. It was also compatible with our systematic review findings that identified an elevated risk of DEB among older Saudi students compared with younger counterparts [29]. A rationale for this distinct pattern is the age-related increase in self-consciousness, including interior and exterior aspects of self, associated with concerns of social acceptance and self-presentation [50]. For that, mid-adolescence (14–17 years) is a critical stage for females to be at a significant risk for emotional and dietary disturbances [51]. However, it is important to acknowledge that the literature findings were not conclusive in relation to age, as most studies were focused on comparing adolescents and young adults rather than the full age spectrum of adolescents [24].

Our results from this cross-sectional survey that the BMI category was not a significant predictor of DEB appeared inconsistent with previous evidence, suggesting that higher BMIs are a potential risk factor for DEBs. However, in our analysis, BMI was significantly associated with dieting subscale scores and inversely associated with oral control subscale scores. Despite non-significant findings, the results indicated that students with obesity were at almost double the risk of DEB compared with students who were underweight. Significant BMI with dieting subscale scores indicated that students with overweight and obesity tended to diet and restrict food and were preoccupied with thinness more than their counterparts with normal and underweight BMI [45,52]. Moreover, BMI was inversely associated with oral control subscale scores, indicating that students with lower BMI had more self-control over eating and experienced higher levels of pressure from others to gain weight compared with students with overweight and obesity [45,52]. Studies from Saudi Arabia and Arab countries have confirmed a significant connection between BMI and DEB risk with adolescents living with overweight and obesity, showing a higher risk of DEB compared with normal BMI adolescents [12,33,53]. It was noted that, regardless of gender, adolescents with obesity had a two-to-three times higher likelihood of having a DEB than adolescents who were non-obese [33]. In Kuwait, when comparing students who were obese and non-obese, both genders of students with obesity were at a twofold-increased risk of developing DEB [53]. Similarly, studies from the UK and the US reported significantly more DEB in groups that were overweight and obese [54,55].

School regions in Riyadh did not appear to be a strong predictor of DEB, with only two schools showing significant variations—the 213 Intermediate School in the north and the 40 Intermediate School in the west. Due to the lack of literature that focuses attention on the epidemiology of DEB and region, explaining differences can be complex [43]. Hay and Mitchison highlighted in their scoping review that urbanisation can be associated with increasing DEB risk when it is linked with other sociodemographic factors [31]. Miller and Pumariega confirmed that cultural beliefs can be major contributors to the development of DEB and suggested that cultural change may be related to an increased sensitivity to disordered behaviours, particularly when physical appearance concepts are implicated [56].

All individual DEBs, including binging, induced vomiting, consuming laxatives, exercising and losing weight, were significantly higher among students who scored 20 or more on the EAT-26. Among the high-risk group, binging was reported once a day or more by 8% of students and 6.5% reported binging two to six times a day. About 6% engaged in induced vomiting once a day or more and 26% reported losing 9 kg or more in the last 6 months. Comparable rates were presented in other studies using the same test in Saudi Arabia and Western countries [24,41]. A Saudi cross-sectional study addressing the prevalence of DEB among 393 high-school female students aged 15 to 20 years in Al-Madinah City, Saudi Arabia, identified that 25.2% practiced binging behaviour, followed by induced vomiting (8.7%) and the use of laxatives (6.1%) [24]. Another study evaluated the prevalence of such disorders among female students aged 12–18 years from schools in the Western population, particularly in Canada [41]. Out of 2483 adolescent females, binging was reported by 15% of students, induced vomiting was reported by 8.2% and diet pills by 2.4% [41].

While social, political and economic issues that impact the prevalence of disordered eating have received a lot of attention, there has been relatively little focus on eating disorder prevention in eating disorder research [57]. The increased prevalence of DEB among Saudi adolescents confirms the need for developing primary preventive strategies in intermediate and secondary schools to limit such behaviours at vulnerable ages.

## 5. Limitations

Due to the cross-sectional design, causal inferences cannot be made. This study was characterised by collecting data at one specific point in time using a self-reported test; no interview or other diagnostic tools were used to confirm the risk. More information on school characteristics and participants’ socioeconomic characteristics, including parental income and education and individual psychological and lifestyle factors, could have explained some of the differences by school. However, this was not collected, as this was not the primary aim of this study. Non-response bias among students who were overweight and obese was also possible due to shame, denial or secrecy [58].

## 6. Conclusions

To our knowledge, this is the first study exploring the prevalence of DEBs and associated predictors in intermediate and secondary schools in Riyadh using EAT-26. About 30% of female school adolescents in Riyadh reported scores in EAT-26 that identified them to be at a high risk of developing a DEB. This suggests it is imperative to increase adolescents’ awareness on healthy eating practices. Early detection and diagnosis could play a major role in limiting complications and avoiding the progression to an ED. The EAT-26 test can be applied in schools as a preliminary screening tool for early detection and referral to promptly deliver the required medical intervention. The nature of the relationship between BMI and DEB requires further clarification; this could include qualitative research to investigate the complexities of how current BMI is related to DEB, as this is likely to be a bi-directional relational rather than a simple causal association in which current BMI is the cause for DEB or vice versa. Further research is needed to explore major determinants and contributors causing DEB in the Saudi population. This would support the development, implementation and evaluation of specific preventive programs related to body weight and proper eating practices.

## Figures and Tables

**Table 1:** Demographic characteristics of the study population.

Variable	*n*	%
Age (*n* = 416)		
12–13 years	172	41.3
14–15 years	95	22.8
16–17 years	121	29.1
18–19 years	28	6.7
Mean ± SD	14.56 ± 1.89	
School (Region)		
128 Secondary School (North)	63	15.1
213 Intermediate School (North)	135	32.5
59 Secondary School (East)	117	28.1
40 Intermediate School (West)	101	24.3
Grade		
Intermediate Grade 7	174	41.8
Grade 8	55	13.2
Grade 9	7	1.7
Secondary Grade 10	83	20
Grade 11	41	9.9
Grade 12	56	13.5
Weight Mean ± SD	56.39 ± 13.71	
Height Mean ± SD	157.8 ± 6.32	
BMI Mean ± SD	22.56 ± 4.92	
Underweight	48	11.5
Normal	211	50.7
Overweight	102	24.5
Obese	55	13.2

**Table 2:** Distribution of EAT-26 scores by age, BMI and school.

	EAT-26 Categories		*p*-Value	OR	95% CI
	High Risk (EAT-26 ≥ 20)	Low Risk (EAT-26 < 20)			
Age Younger (12–15 years)	74 (27.7%) ^1^	193 (72.3%)	0.017 *	3.087	1.228–7.760
Older (16–19 years)	49 (32.9%)	100 (67.1%)			
BMI Underweight ^$^	11 (22.9%)	37 (77.1%)	0.322		
Normal	62 (29.4%)	149 (70.6%)	0.430	1.349	0.641–2.837
Overweight	29 (28.4%)	73 (71.6%)	0.433	1.382	0.616–3.103
Obese	21 (38.2%)	34 (61.8%)	0.080	2.194	0.911–5.286
School (Region) 128 Secondary School (North) ^$^	18 (28.6%)	45 (71.4%)	0.185		
213 Intermediate School (North)	40 (29.6%)	95 (70.4%)	0.047 *	3.109	1.016–9.509
59 Secondary School (East)	34 (29.1%)	83 (70.9%)	0.607	1.202	0.596–2.424
40 Intermediate School (West)	31 (30.7%)	70 (69.3%)	0.047 *	3.071	1.016–9.282

^1^ Results are explained in *n* (%). ^$^ Reference group. * Significant *p* value.

**Table 3:** Students’ responses to EAT-26 dieting subscale, oral control subscale and bulimia and food preoccupation subscale.

Students’ Responses to EAT-26 Dieting Subscale		
Q	Dieting Scale Questions	Mean ± SD
1	I am terrified about being overweight	1.07 ± 1.25
6	Aware of the calorie content of food that I eat	0.64 ± 1.06
7	Particularly avoid food with a high carbohydrate content (bread, rice, potatoes, etc.)	0.28 ± 0.71
10	Feel extremely guilty after eating	0.48 ± 0.99
11	Am preoccupied with a desire to be thinner	1.06 ± 1.26
12	Think about burning up calories when I exercise	1.33± 1.31
14	Am preoccupied with the thought of having fat on my body	0.67 ± 1.09
16	Avoid foods with sugar in them	0.32 ± 0.72
17	Eat diet foods	0.35 ± 0.79
22	Feel uncomfortable after eating sweets	0.62 ± 1.06
23	Engage in dieting behaviour	0.26 ± 0.73
24	Like my stomach to be empty	0.53 ± 1.0
26	Enjoy trying new rich foods	0.74± 0.99
Students’ Responses to EAT-26 Oral Control Subscale		
Q	Oral Control Scale Questions	Mean ± SD
2	Avoid eating when I am hungry	0.44 ± 0.87
5	Cut my food into small pieces	0.42 ± 0.89
8	Feel that others would prefer if I ate more	0.59 ± 1.05
13	Other people think that I am too thin	0.92 ± 1.24
15	Take longer than others to eat my meals	0.72 ± 1.11
19	Display self-control around food	1.37± 1.23
20	Feel that others pressure me to eat	0.71 ± 1.16
Students’ Responses to EAT-26 Bulimia and Food Preoccupation Subscale		
Q	Bulimia and Food preoccupation Scale Questions	Mean ± SD
3	Find myself preoccupied with food	0.38 ± 0.79
4	Have gone on eating binges where I feel that I may not be able to stop	0.20 ± 0.63
9	Vomit after I have eaten more	0.14 ± 0.52
18	Feel that food controls my life	0.50 ± 0.93
21	Give too much time and thought to food	0.32 ± 0.83
25	Have the impulse to vomit after meals	0.22 ± 0.68

**Table 4:** Responses to behavioural questions based on the past six months.

Behavioural Questions	(*n*, %)					
	Never	Once a Month or Less	2–3 Times a Month	Once a Week	2–6 Times a Week	Once a Day or More
QA: Gone on binges where you feel that you may not be able to stop?	258, 62%	81, 19.5%	33, 7.9%	13, 3.2%	15, 3.6%	16, 3.8%
QB: Ever made yourself sick (vomited) to control your weight or shape?	358, 86%	22, 5.3%	7, 1.7%	10, 2.4%	4, 1%	15, 3.6%
QC: Ever used laxatives, diet pills or diuretics (water pills) to control your weight or shape?	395, 95%	8, 1.9%	5, 1.2%	3, 0.7%	1, 0.2%	4, 1%
QD: Exercised more than 60 min a day to lose or control your weight?	160, 38.5%	83, 20%	51, 12.3%	58, 13.9%	37, 8.9%	27, 6.4%
	Yes			No		
QE: Lost 9 kg or more in the last 6 months.	61, 14.7%			355, 85.3%		

**Table 5:** Correlation of students’ EAT-26 subscales with age and BMI.

	Dieting Scale	Oral Control Scale	Bulimia and Food Preoccupation Scale
Age	0.069	−0.069	0.075
BMI	0.347 *	−0.299 *	0.074

* *p*-value is (*p* < 0.001).

## Data Availability

Data are contained within the article and supplementary materials.

## Supplementary Materials

The following supporting information can be downloaded at: https://www.mdpi.com/article/10.3390/nu16020281/s1, File S1. Sample Size Calculation using EpiInfo.
