# Supplementary material for: The Prevalence of Disordered Eating Behaviours (DEBs) among Adolescent Female School Students in Riyadh, Saudi Arabia: A Cross-Sectional Study
Source: Nutrients. 2024 Jan 17;16(2):281. doi: 10.3390/nu16020281 (PMC10818681; doi:10.3390/nu16020281)
Supplement: Supplementary file 1 [file nutrients-16-00281-s001.zip › nutrients-2811624-supplementary.pdf]

File S1. Sample Size Calculation using EpiInfo.

Sample Size for Frequency in a Population

Population size(for finite population correction factor or fpc)(*N*):	1000000
Hypothesized % frequency of outcome factor in the population (*p*):	16%+/-5
Confidence limits as % of 100(absolute +/- %)(*d*):	5%
Design effect (for cluster surveys-*DEFF*):	1

Sample Size(*n*) for Various Confidence Levels

ConfidenceLevel(%)	Sample Size
95%	207
80%	89
90%	146
97%	254
99%	357
99.9%	582
99.99%	814

Equation

Sample size $n = [DEFF*Np(1-p)]/ [(d^2/Z^2_{1-\alpha/2}*(N-1)+p*(1-p)]$

Results from OpenEpi, Version 3, open source calculator--SSPropor
